# Enhancing skill learning with dual-user haptic feedback: insights from a task-specific approach

**DOI:** 10.3389/frobt.2023.1286282

**Published:** 2023-11-23

**Authors:** Yao Zhang, Olyvia Wang, Yanqing Wang, Mahdi Tavakoli, Bin Zheng

**Affiliations:** ^1^ Surgical Simulation Research Lab, Department of Surgery, University of Alberta, Edmonton, AB, Canada; ^2^ Scopemedia Inc., Vancouver, BC, Canada; ^3^ Electrical and Computer Engineering Department, University of Alberta, Edmonton, AB, Canada

**Keywords:** haptic interface, skill acquisition, task performance and analysis, simulation, hands-on training

## Abstract

**Introduction:** This study was to examine whether inter-user haptic feedback would have a differential impact on skill acquisition based on the nature of the surgical task involved. Specifically, we hypothesized that haptic feedback would facilitate target orientation more than cutting tasks in the context of laparoscopic surgery.

**Methods:** Ten novice participants were recruited and assigned to one of two training groups. Each group underwent six half-hour training sessions dedicated to laparoscopic pattern-cutting tasks. In the haptic group, five participants received expert guidance during the training sessions, whereas the remaining five participants in the control group engaged in self-practice. All trials were recorded on video, enabling a comparative analysis of task performance between the participants’ left hand (target manipulation) and right hand (cutting task). Additionally, the number of haptic feedback instances provided to the trainees in the haptic group was recorded.

**Results:** Practice led to a reduction in total task time, grasping time, and cutting errors. However, no significant differences were observed between the two training groups, except for the grasping time, where haptic feedback significantly reduced the grasping time compared to the control group. Moreover, the frequency of haptic feedback instances provided to the trainees was notably higher for the grasping than for the cutting task.

**Discussion:** Our study suggests that haptic feedback has a more substantial impact on orientation tasks than on cutting tasks in laparoscopic surgery training. However, we acknowledge that a larger sample size would provide a more robust evaluation of this effect.

## 1 Introduction

Haptic refers to the sense of touch, particularly in relation to the perception and manipulation of objects through the senses of touch and proprioception (Westebring – van der Putten et al., 2008). In surgery, wearing gloves can diminish touch sensations, while using tools, especially power tools with vibration, can disturb proprioceptive feedback (Westebring – van der Putten et al., 2008). Our previous study showed that diminishing surgeons’ touch sensations by wearing double gloves and employing long-shaft instruments downgraded surgical performance (Zhang et al., 2022a).

Can we create augmented haptic feedback to improve surgical performance? This is challenging in real surgery due to the limitations of current technology (Webb and Pentlow, 1993; Westebring – van der Putten et al., 2008; Hardison et al., 2017; Chainey et al., 2021). Nonetheless, there are emerging robotic systems designed to provide haptic assistance to surgeons in the operating room (Zhang et al., 2022b), signifying a pivotal direction for the future evolution of surgery. Outside the operating room, artificial sensations of touch and resistance from an illusory substance can be generated within the virtual world using haptic devices, such as the Geomagic PHANTOM Omni (Geomagic Inc., Morrisville, NC, United States) (Basdogan et al., 2004; Samur et al., 2012). Haptic devices generate force based on varying texture and tissue properties, subsequently providing feedback to a human user. Being able to feel virtual objects through haptic feedback has been proven to have positive impacts on physicians’ performance when they interact with the virtual world. Haptic feedback could guide arterial catheterization, promote the physician’s navigation, identification, and characterization of tissues, as well as enhance the surgeon’s performance while taking a biopsy from a patient (Gobbetti et al., 2000; Konstantinova et al., 2013; Tercero et al., 2013). However, the role of artificial haptic feedback in skill acquisition remains debatable (Zheng et al., 2006; Pinzon et al., 2017).

Besides connecting a human user to virtual objects via artificial haptics, it is possible to deliver haptic feedback from one individual to another by connecting multiple haptic devices (Chellali et al., 2012; Heidari et al., 2019; Motaharifar et al., 2019). In the Surgical Simulation Research Lab (SSRL), we connected four Phantoms to build a *dual-user haptic training system*, which allowed the movements from both hands of one individual to be transmitted to the corresponding hands of another person (Pinzon et al., 2016). We can capture an expert’s manipulation and, through haptic feedback, deliver their movements to the novice in real-time. In this way, trainees will not only be able to see what the expert has done, but also perceive the movements. Such a dual-user haptic training model has created a new way of hand-to-hand training, claiming benefits for skill training outside of healthcare. In the realm of robot skill learning, systems have been developed that enable human operators to rectify the behavior of autonomous robots (Si et al., 2021; Si et al., 2022). Drawing from this idea, we devised a method allowing experts to provide instantaneous guidance to novices. However, when applying this concept to surgical training in our previous study, we were not able to quantify the advantages of receiving an expert’s haptic feedback in learning laparoscopic tasks (Lu et al., 2021). We attribute the reasons to the periodic nature of feedback provision and the way of measuring surgical performance. In this project, we set up an experiment that allowed trainees to receive instant haptic guidance from an expert continuously and measured the impact of haptic feedback for two different types of surgical tasks: an orientation task and a cutting task.

Our study distinguishes itself by categorizing the learning tasks into “fast executive subtasks” (simple feedforward tasks) and “orientation-type subtasks” (feedback sensitive). Limited by current technology for haptic rendering, haptic feedback transmitted via the dual-user haptic training platform mainly focused on movement distance and direction, rather than touch sensation and manipulation (Pinzon et al., 2017). This type of haptic information is expected to have a greater influence on a trainee’s movement direction and distance (navigation) than on their rapid hand actions, such as cutting with scissors, because it demands a higher degree of movement precision and spatial awareness, necessitating ongoing movement adjustment through both visual and haptic feedback loops. We anticipated that haptic feedback might have different impacts on different types of tasks. Previous literature supports the notion that tasks involving grasping require more haptic guidance than those centered around reaching (MacKenzie and Iberall, 1994; Zheng and MacKenzie, 2007). In the context of our study, tasks related to fabric orientation closely align with grasping movements, while scissors cutting resembles a reaching movement, predominantly guided by feed-forward processes. While fast executive tasks might not be sensitive to haptic feedback, the orientation-type tasks might respond well to it.

We therefore hypothesize that continuous between-person haptic feedback would facilitate the learning of laparoscopic skills more than self-learning, quantified by a shorter target orientation time rather than a cutting time. Differentiating the impact of haptic feedback between these tasks can guide us in developing effective haptic-driven surgical training protocols. Instructors can be informed to deliver haptic feedback during the feedback-sensitive phase to potentially enhance training efficacy.

## 2 Materials and methods

### 2.1 Apparatus

**FIGURE 1:**
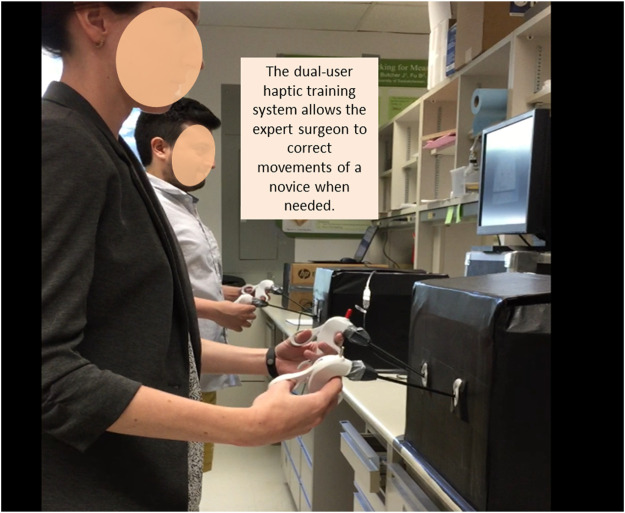
Setup of the dual-user haptic training system. Each of the two training boxes houses two haptic feedback devices (Phantom), connected to laparoscopic instruments. Connection of these two devices was describe elsewhere (Pinzon et al., 2017). Participants were asked to perform a fabric cutting task inside the boxes, using a laparoscopic grasper in their left hand and scissors in their right. The expert delivers haptic feedback to the novice when needed.

The research was performed in the Surgical Simulation Research Lab at the University of Alberta, where four Phantom Omni devices were connected (Figure 1) (Pinzon et al., 2017). Each Phantom has 6-DOF (degrees of freedoms) position input and 3-DOF haptic feedback in the *x*-*y*-*z* directions, allowing maximum exertable force at 3.3 N. The four Phantoms were connected in parallel using Simulink (Mathworks, Palo Alto, CA), a graphical programming environment designed for creating simulation tasks based on MATLAB. Details about this dual-user haptic training system can be found in our previous paper (Pinzon et al., 2017; Lu et al., 2021). Briefly, the system enables the translation of hand movements from the instructor side to the trainee side.

**FIGURE 2:**
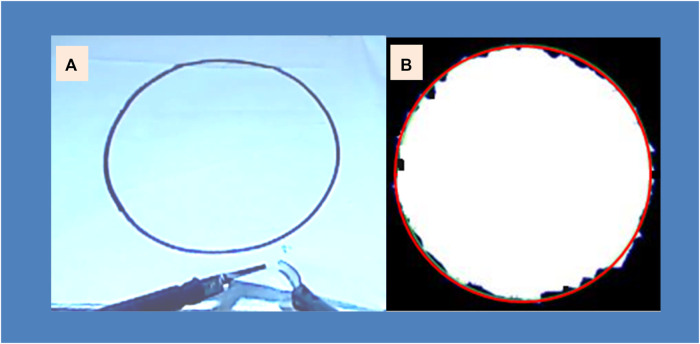
**(A)** The pattern cutting task executed using laparoscopic graspers and scissors. **(B)** Assessment of cutting accuracy, determined by measuring the deviation between the target red circle and the actual cut edge.

To enable the attachment of laparoscopic instruments, the stylus of each Phantom was modified. Two instruments, a grasper on the left-hand side and laparoscopic scissors on the right-hand side, were inserted into a laparoscopic training box. A piece of fabric with a pre-defined circle drawn at its center was placed at the bottom of the training box (Figure 2). Participants were instructed to cut the circle from the fabric. Video recordings of the task were captured by a webcam and displayed on a TV monitor located 75 cm in front of the participant. The same video was simultaneously displayed on a second TV monitor at the trainee’s side (Figure 1). During the task, both the instructor (an expert laparoscopic surgeon) and trainee viewed the same video. The instructor provided haptic feedback to adjust the trainee’s movements based on their own judgment.

### 2.2 Participants

Ten right-handed university students, with normal or correct-to-normal vision and no history of motor control issues, were recruited for the study. Participants were chosen based on their availability and commitment to the duration of the study, and a paramount criterion in our selection process was the naivety of participants to surgical practices. We aimed to recruit participants with no previous exposure to surgical training to ensure that the skill learning observed was a direct result of the training implemented. The research was approved by the University of Alberta Health Research Review Board, and all participants provided written consent prior to participating in the experiment.

### 2.3 Task and procedure

A random code was generated in Microsoft Excel with an equal probability of generating 1 vs 2. Participants were randomly assigned to one of two groups: haptic feedback (code 1) or self-learning control (code 2). Each group completed six 30-min training sessions over a 2-week period.

In the control group, participants were given only a first instructional video and then practiced the cutting task without further guidance. In the haptic feedback group, participants watched the instructional video. When they started practice, an expert instructor provided haptic guidance during training sessions, but verbal communication was not allowed.

All participants were required to perform the pattern cutting task, which involved cutting out a pre-drawn circle in the center of a piece of fabric using laparoscopic graspers and scissors (Figure 2A). The grasper was held in the left hand of the trainee, while the scissors were held in the trainee’s right hand. This task was adapted from one of the tasks listed in the Fundamentals of Laparoscopic Surgery (Peters et al., 2004).

### 2.4 Measure of performance

Each completed trial performed by a participant was video recorded and used for extracting performance data. The duration of the *total task time* was determined as the interval between the initial contact of the laparoscopic instruments with the fabric and the completion of fully cutting out the circle from the fabric. This measures the overall efficiency of the participant’s task. As surgical tasks often need to be performed under time constraints, it is crucial to understand how haptic feedback might influence the speed of task completion.

The *total grasping time* encompassed the overall duration of the grasper’s manipulations on the target, involving a sequence of deliberate and gradual movements such as biting and rotating the fabric to reposition it for cutting. This specifically measures the duration required for the grasper to orient the fabric, reflecting the more time-consuming and feedback-reliant tasks. The *total scissor cutting time* accounted for the combined duration of linear and rapid cutting movements, each starting from the opening of the scissor jaws and ending with their closure. This metric captures the cutting action performed by the scissors, representing the quick-executed aspects of the surgical task. It should be noted that the total task time was not always equivalent to the sum of the total grasping time and total scissor time. Participants often performed grasping and cutting simultaneously, and there were instances where neither the graspers nor the scissors were actively engaged in the performance.

The frequency of expert corrections made to the trainee’s movements was recorded and reported as the *number of haptic feedback* instances provided to correct either the grasper or scissor movement.

The last measure was *cutting accuracy*, which was assessed by calculating the deviation between the actual cutting line and the predefined circle line. By measuring cutting accuracy, we aim to gauge the extent to which haptic feedback can enhance a trainee’s precision. At the end of each valid cutting performance, the cut-out circle was scanned, and the area (mm^2^) was reported to describe the deviation of the actual cut from the predefined line (Figure 2B).

### 2.5 Statistical analysis

The above performance measures were analyzed using the *SPSS 22.0* (IBM, Chicago, IL), employing a 2 (group) × 6 (training session) mixed ANOVA with repeated measurements on the second factor. The number of haptic guidance instances given by the expert was compared between the two tools and the six training sessions. Mean ± standard deviation was used to report the group differences, while *p* < 0.05 was considered to indicate significant differences. In cases where necessary, *post hoc* analysis was conducted using *Bonferroni* analysis.

## 3 Results

The test on the main factor of training group showed no significant differences between the haptic feedback and the self-learning groups in terms of the total task time (*p* = 0.570), the scissor cutting time (*p* = 0.793) and errors (*p* = 0.161) (Table 1). However, the total grasping time was significantly different (*p* = 0.046) between the two training groups, with the control group taking longer time (250.4 ± 63.3 s) than the haptic group (211.0 ± 51.9 s).

**TABLE 1:** Statistic outputs on task performance over six training session between two different training group.

		Day 1		Day 2		Day 3				
		Session 1	Session 2	Session 3	Session 4	Session 5	Session 6	P (group); η^2^	P (session); η^2^	P (interaction); η^2^
Total time	Control	403.0 ± 59.0	373.6 ± 107.3	323.8 ± 45.2	333.2 ± 75.2	278.2 ± 44.4	306.4 ± 57.9	0.570; 0.04	0.001; 0.52	0.533; 0.08
(ms)	Haptic	415.2 ± 39.0	366.6 ± 65.1	311.4 ± 37.6	310.8 ± 59.7	296.6 ± 56.4	242.8 ± 39.4			
Grasper time	Control	310.6 ± 59.1	276.8 ± 74.1	237.2 ± 28.0	258.4 ± 69.8	203.2 ± 45.7	216.4 ± 48.5	0.046; 0.41	0.000; 0.57	0.671; 0.06
(ms)	Haptic	283.6 ± 39.3	245.8 ± 42.4	201.2 ± 19.4	197.2 ± 28.8	184.2 ± 27.4	153.8 ± 27.3			
Scissors time	Control	98.6 ± 24.0	105.6 ± 42.0	111.8 ± 23.7	81.6 ± 16.8	98.1 ± 23.2	100 ± 22.2	0.793; 0.01	0.290; 0.14	0.371; 0.12
(ms)	Haptic	125.1 ± 17.5	108.2 ± 21.6	96.8 ± 19.3	95.2 ± 15.4	95.2 ± 40.7	85 ± 14.7			
Error (mm^2^)	Control	27.3 ± 7.8	22.6 ± 3.0	21.1 ± 4.4	17.7 ± 7.0	15.8 ± 4.5	19.2 ± 3.0	0.161; 0.23	0.000; 0.58	0.588; 0.07
	Haptic	26.0 ± 6.1	20.5 ± 3.4	14.9 ± 3.2	16.9 ± 5.3	13.1 ± 2.0	14.7 ± 4.4			

**FIGURE 3:**
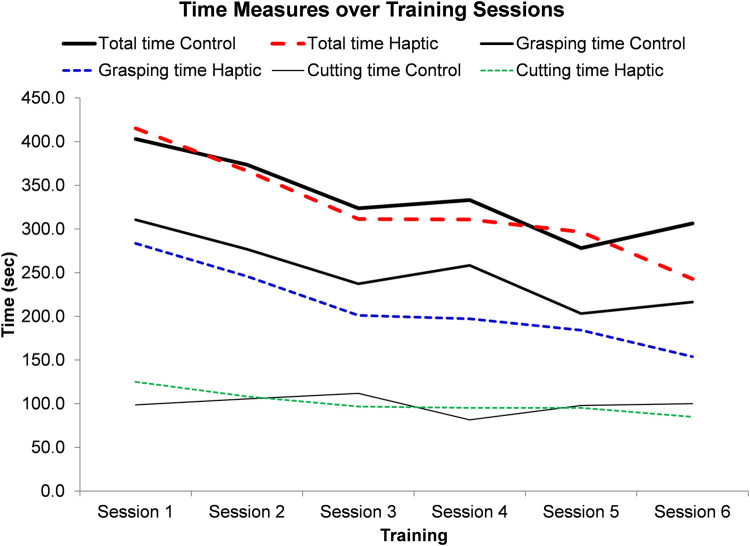
Time measures of two training groups across six training sessions.

The test on the main factor of training session showed significant differences in terms of the total task time (*p* = 0.001), the total grasping time (*p* < 0.001) and error (*p* < 0.001) (Table 1), but not on the total scissor cutting time (*p* = 0.290). Specifically, the total task time, the total grasping time and errors reduced as the practice increased (Figure 3). No significant interaction effect was reported between the training group and training session (Table 1).

**FIGURE 4:**
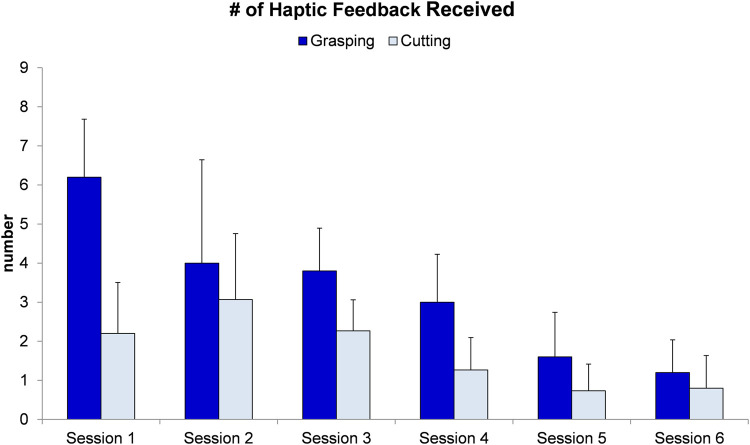
Number of haptic guidance given to two instruments across six training sessions.

We found the expert delivered significantly more haptic feedback to the trainees during the grasping (3.3 ± 2.2) than the cutting task (1.7 ± 1.3; *p* < 0.001). We also found that number of haptic feedbacks was significantly reduced as the practice (training sessions) increased (Session 1: 4.2 ± 2.5; Session 2: 3.5 ± 2.1; Session 3: 3.0 ± 1.2; Session 4: 2.1 ± 1.3; Session 5: 1.2 ± 1.0; Session 6: 1.0 ± 0.8; *p* < 0.001. Figure 4).

## 4 Discussion

Our study demonstrates that practice over time resulted in skill improvement as indicated in Figure 2. Both the haptic and control groups exhibited enhancements in the total task time, the grasping (target orientation) time, and cutting accuracy over the course of six training sessions. However, the addition of haptic feedback to laparoscopic training did not yield significant benefits in terms of total task time or error reduction compared to self-learning. The only notable advantage observed between the two training groups was in the total grasping time, where receiving haptic feedback from an expert facilitated faster grasper movements. We would like to recap that the total task time was not always equivalent to the sum of the total grasping time and total scissor time. As we mentioned before, participants often performed grasping and cutting simultaneously, and there were instances where neither the graspers nor the scissors were actively engaged in the task performance. This could potentially explain why reducing the total grasping time did not result in a decrease in total task time.

The circle-cutting task used in our study required a high level of bimanual cooperation using intricate surgical instruments (Peters et al., 2004; Cassera et al., 2012). In the laparoscopic setting, two instruments were inserted into the training box (surgical site) through pre-defined entrance ports (Supe et al., 2010). Compared to open surgery, participants performing this laparoscopic task had limited degrees of freedom in manipulating the scissors. Instead, the grasper was capable of biting on and orientating the piece of fabric, creating a proper position, and stretching force on the fabric for the scissors to cut. Manipulating the grasper was more important than controlling the scissors for successful pattern cutting performance. Our data indicated that haptic feedback provided by the expert mainly focused on assisting grasper movement rather than scissors’ movement (Figure 4). As a result, the grasping time reduced significantly in the haptic compared to the control group, even though the total task time did not reach a significant level of difference. This may be due to the small sample size (partial eta η2 was 0.04).

Our findings indicate that providing between-person haptic feedback focused on orientation-based subtasks leads to improved learning outcomes for trainees compared to feedback focused on fast executive subtasks like cutting. This distinction arises from the limitations of the current dual-user haptic training model. The transmission of information via the Phantom devices requires time, and human operators in the loop also require time to receive and deliver feedback to one another (Westebring – van der Putten, et al., 2008; Chellali et al., 2012; Escobar-Castillejos et al., 2016). Based on our results, it is evident that the haptic feedback provided by the expert proved more beneficial for the slower subtask involving orientation, as opposed to the faster subtask involving linear motion. Our findings offer valuable insights, especially for optimizing the design of surgical training protocols. For instance, by understanding at which stage a learner requires haptic guidance, we can provide the necessary feedback, either through an expert in the current setting or from a robotic system in future projects.

Another important aspect to consider is the availability of haptic feedback delivery. In our previous study, trainees received periodic haptic feedback from an expert, which involved passively receiving the expert’s movement for one trial followed by self-practicing for a few trials (Lu, Wang, Sanchez, Kathrada, Tavakoli and Zheng, 2021). This protocol did not allow for instant feedback delivery to guide or correct the trainees’ movements. In contrast, in our current study, we allowed participants to receive instant haptic feedback, which we believe led to the significant improvements in task time and accuracy observed across the subsequent training sessions. This finding aligns with recent research on surgical training, which has shown that expert feedback provided in a parallel, continuous haptic setting can facilitate the performance of novice operators experiencing moments of difficulty (Escobar-Castillejos, Noguez, Neri, Magana and Benes, 2016; Motaharifar, Taghirad, Hashtrudi-Zaad and Mohammadi, 2019). We suggest that incorporating this type of haptic feedback delivery into laparoscopic training programs may yield noticeable benefits for skill learning.

Our study’s findings could be applied to general surgical settings. In our previous research, we have decomposed common laparoscopic procedures into fundamental human movements, including reaching, grasping, orientation, and cutting (Cao et al., 1999). These basic movements, including tissue orientation, are integral components of various surgical subtasks, such as grasping tissues, dissection, and suturing. It is important to note, however, that while haptic feedback can be beneficial in many contexts, there may be certain surgical scenarios where it is less critical. For example, in tasks that are predominantly visually guided and require less tactile discrimination, the reliance on haptic feedback may be reduced. Understanding when and where haptic feedback is most beneficial allows for a more targeted and effective training approach.

One limitation of our current study was the nature of the participants. We recruited students without prior surgical experience to examine their learning processes under the influence of haptic feedback. Nevertheless, we acknowledge that our sample may not fully encapsulate the learning trajectory of in-training practitioners. In future studies, it will be essential to extend our participant pool to include surgical residents. This will provide a more representative sample of the surgical trainee demographic, allowing us to validate and potentially generalize our findings to the broader community of in-training practitioners. We also noticed that the small sample size may limit our power in testing our hypothesis. Therefore, we will recruit surgical residents and enlarge our sample size to increase statistical power in future studies. We view our work as a foundational step that will pave the way for subsequent, more comprehensive studies, and we believe further research with larger cohorts in this topic would be valuable.

It is also important to note that the circle-cutting task used in our study was a simple task and does not represent an actual surgical procedure performed in the operating room. Therefore, caution will be needed when applying our findings to a clinical setting. Future studies should explore the performance of surgeons performing real surgical tasks that require complex eye-hand, bimanual, and team coordination, which may yield different outcomes.

While our primary focus and dataset originated from a surgical setting, we believe that our findings hold potential for broader applications. Tasks that are complex, compound in nature, and require continuous adjustment based on environmental constraints are likely to benefit from the insights provided by our research. This includes activities such as loading delicate equipment, orientating objects in constrained spaces, and grasping irregularly shaped items, which can be found in various domains outside healthcare.

We recognize the necessity for carefulness in extending the findings of this research to wider contexts. In scenarios where tasks necessitate rapid, impulsive movements, such as in reaching for or striking an object, the necessity for feedback may be diminished. This is because these types of movements are often guided by pre-planned motor commands, where there is limited time for sensory feedback to be integrated and influence the action. On the other hand, tasks that require fine-tuned adjustments and instant adaptations to environmental constraints heavily rely on feedback. Examples of such tasks include grasping an irregularly shaped object, loading delicate equipment, or orientating tools in a constrained space. In these situations, haptic and visual feedback become invaluable as they enable the performer to make precise modifications in real-time, ensuring accuracy and safety. To go beyond the current limitations and enhance the generality of our findings in future work, we could diversify the range of tasks examined, incorporate a broader spectrum of actions, and explore the applicability of our findings in different settings and domains.

## 5 Conclusion

In conclusion, the dual-user haptic training system provides a promising new avenue for transmitting haptic feedback in surgical training. Our study indicated that the use of haptic feedback in laparoscopic training can improve performance in certain aspects of the task, specifically in target orientation. Our findings suggest that haptic feedback superimposed on the user’s motions during orientation-type subtasks may yield better learning outcomes than on fast executive subtasks such as a cutting. Receiving instant haptic feedback during moments of performance difficulty was found to be beneficial to trainees’ learning process. The small sample size and the use of a simple task limit the generalizability of our findings, and future studies involving surgical residents and more complex tasks are needed.

## Data Availability

The raw data supporting the conclusion of this article will be made available by the authors, without undue reservation.
